# A scientific methodology course for advanced medical students: an eight-year perspective

**DOI:** 10.12688/mep.19171.1

**Published:** 2022-07-06

**Authors:** Silvina Bartesaghi, Gastón Garcés, Enrique Barrios, Rafael Radi

**Affiliations:** 1 Departamento de Bioquímica and Centro de Investigaciones Biomédicas (CEINBIO), Facultad de Medicina, Universidad de la República, Montevideo, 11800, Uruguay; 2 Departamento de Educación Médica, Facultad de Medicina, Universidad de la República, Montevideo, 11800, Uruguay; 3 Departamento de Métodos Cuantitativos, Facultad de Medicina, Universidad de la República, Montevideo, 11800, Uruguay

**Keywords:** Medical Education, Scientific Methodology, Scientific Training, Uruguay

## Abstract

**Background: **Exponential increases in the development of medical knowledge, the expansion of areas where medicine develops its activities, the emergence of new pathologies ( *e.g.,* COVID-19), novel diagnostic methods and therapeutic strategies, together with the appearance of multiple communication and information technologies, determined that the education of future physicians required targeted training in scientific methodology.

**Methods: **The design and execution of a course in scientific methodology in the *curriculum *of Facultad de Medicina, Universidad de la República, Uruguay, is described. The course is carried out at an advanced stage of the medical studies for all the students, in which they develop a 10-month research project supervised by the medical school faculty. Students undergo all stages of a research endeavor: generation of hypothesis, elaboration of a research protocol, submission to the Research Ethics and Animal Welfare Committees, data recollection, analysis, interpretation and publication of the results.

**Results: **The course is undertaken at the Facultad de Medicina, Universidad de la República, Uruguay, the main university of the country, with high numbers of students enrolled. The course involves the participation of 600 students and up to 300 professors *per* year, which implies a huge institutional effort

**Conclusions: **The scientific methodology course resulted in one of the most important incorporations of the current 2008 *curriculum*. Local students, faculty and international evaluators have qualified this activity as an educational breakthrough, being a gratifying and productive experience. The course represented the first exposure of medical students to the research methodology, scientific literature and publication rules, and emphasized the dynamic nature of medical knowledge within modern medical education. Moreover, for some students it constituted the onset of academic research careers. An additional positive outcome was the reactivation of some faculty research projects, in a way that largely exceeded the boundaries of the course.

## Introduction

There is a permanent demand in medical schools to provide a strong background on scientific methodology and research to the future physicians ^1^. The exponential increase of information in molecular medicine, genetic engineering and biotechnology, and the continuous evolution of the paradigms of evidence-based and personalized medicine, fosters a focused training of medical students in scientific aspects of the medical sciences. Therefore, innovations in the medical *curriculum* require the provision of methodological tools and to stimulate a “mindset” for future physicians with the ultimate goal of improving medical practice. The organization of these innovations needs to be adapted, among other factors, to the specific institutional capacities and the number of students.

Universidad de la República in Uruguay, being public and the largest university of the country, has seen a significant increase in students in the last decade, overall representing around 80% of university students in the whole country ^2^. The number of active students (those who have presented activity in the last two years), increased from 81,774 in 2008 to 139,830 students in 2019 ^2^. In 2019, 18,549 students were incorporated to Universidad de la República ^2^, of which 2,225 started the medical career at Facultad de Medicina ^a^. Every year, close to 500 medical students graduate ( *e.g.,* 483 in 2019) ^2^. Thus, in this context, a specific course of “Scientific Methodology in the Medical Sciences” for advanced medical students was conceived and executed, taking into consideration the described conceptual framework and also the numerosity of students. Now, an initial assessment of the organization, evolution and impact of the course over the last eight years will be provided.

In 1910, Abraham Flexner published an extensive report analyzing the situation of 155 medical schools in the United States and Canada ^3^, in which he discussed the need to incorporate the basic sciences and a strong scientific component to the training of physicians, believing that this would result in better performance during their clinical practice. From the report arises the need to incorporate training of the scientific method into the medical career, through the formulation of problems, generation of hypothesis and the development of a series of well-designed studies to reject or confirm the hypothesis, with the idea that the skills for problem solving can be applied directly to patient care ^4, 5^. One hundred years after the publication of the Flexner Report, several authors analyzed how it resulted in a transformative document that generated the foundations for the teaching of 20 ^th^ century Medicine ^6– 9^. At the end of the 90s, the need to generate a change in the study plans of medical careers was raised in the United States, as established in the report generated by the Council on Graduate Medical Education (COGME) ^10^. In this context there is a current emphasis, in prestigious schools of medicine at the international level, on explicit scientific training in study plans ^11^. Therefore, educational strategies are in full development and are undergoing experimentation, varying in the different academic centers where they are applied.

Although a causal relationship cannot be attributed, recent data suggest that successful early participation in research can influence the long-term scientific activities of clinicians. For instance, Huynh and co-workers described the incorporation of a surgical research program for medical students, and demonstrated that integrating research early in the medical school *curriculum* provides students with fundamental skills needed for academic achievement, and can help them to establish academic careers ^12^. Another recent work published by Waaijer *et al.*, evaluated the scientific activity of medical students, and its effect on scientific activity after graduation ^13^. The authors demonstrated that the students who published during their career were more likely to continue publishing after graduation, being more scientifically productive ^13^. There is a general consensus that training medical doctors in the 21 ^st^ century requires the incorporation of different new skills, including biomedical informatics, information and communication technologies and scientific methodology in addition to clinical skills, in order to cope with exponential increase in medical knowledge ^12, 14, 15^.

**Figure 1.**
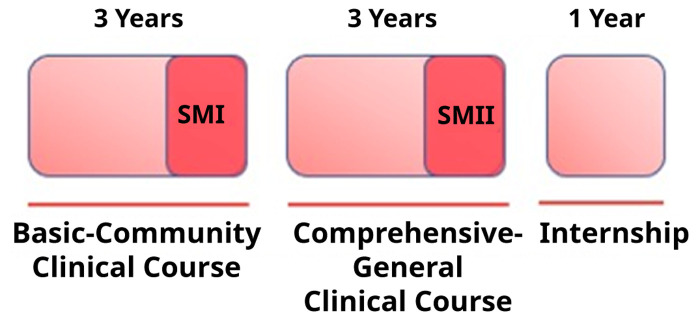
*Curriculum* 2008 organization at Facultad de Medicina, Universidad de la República, and Scientific Methodology courses, SMI and SMII.

In the case of Facultad de Medicina, prior to establishing the Scientific Methodology course described in this work, the past *curriculum* for medical students had only a minor component of research methodology focusing on (basic principles of) biostatistics at the beginning of the career and an approximation of research design in the first year of clinical training ^b^. It is important to note that Uruguayan medical *curriculum* resembles that of European universities and therefore differs from the United States. Indeed, right after high school the medical students initiate a seven-year program (three basic, three clinical, and one year internship) ( Figure 1).

While in 1995 the Asamblea del Claustro ^*c*^ of Facultad de Medicina already referred specifically to the required scientific quality of medical professionals which must " *maintain a critical attitude, based on good scientific training and practice that allows them to analyze, understand and contribute to the resolution of problems related to health in the field in which they operate*” ^16^, it was not until eight years ago that this requirement could be met with specific course content and adequate strategies to effectively enable good scientific training during undergraduate studies. This incorporation was part of the accreditation process at the regional level ^d^ under the MEXA system ^e ^with the generation of a final report in March 2012 ^16^ which allowed the certification of the medical career in November 2012 by ARCU-SUR ^f 17^. One important issue mentioned in the report was the need of advancing scientific training of the medical students, indicating the necessity for improvement ^16^. It is with that objective that within the current *curriculum* of the Medical Doctor degree two courses, Scientific Methodology I and II (SMI and SMII), were established. Although this paper will focus on the SMII course, it is important to point out that the SMI course sets theoretical basis for the second course ( Table 1). 

**Table 1.** Table of contents of the Scientific Methodology Courses I and II.

Biostatistics	Methodological aspects	Bioethics
Scientific Methodology I Course		
Binomial and Poisson distribution	Introduction to epidemiology and epidemiological studies	Ethics of research in human beings. National and international regulations.
Normal distribution	Descriptive studies	Ethical requirements of an investigation in human beings
Diagnostic Procedures	Diagnostic tests	Specific ethical issues
Statistical inference: estimation	Observational analytical studies	Ethical particularities of epidemiological investigations
Risk	Randomized clinical trials	
Statistical inference: hypothesis testing for means	Systematic reviews	
Statistical inference: comparison of proportions	Bases of biomedical literature	
Association tests	Bibliographic searches	
Linear correlation		
Scientific Methodology II Course		
SMI content review	Introduction to research	Ethics of research in human beings
Variance analysis (ANOVA)	Research protocol	Ethical requirements of an investigation in human beings
Linear regression model	Types of research studies	Investigator ethics
Logistic Regression Model	Systematic and narrative reviews	Ethical aspects of bibliographic reviews
Survival analysis and Cox model	Practical aspects of bibliographic search	Ethical aspects of scientific publications
Data analysis tools	Scientific writing and communication- ICTs	
	Graphic presentation of results	

In addition to the Scientific Methodology courses, a series of activities were incorporated into the 2008 *curriculum*, where emphasis was placed on the development of activities leading to much needed integration of basic and clinical aspects in various health-disease processes, analyzing scientific approaches to the problem and with a emphasis on the use of medical databases and bibliography accessible through information and communication technologies (ICT). A series of scientific conferences at the Facultad de Medicina were also formalized since 2013, with local and international speakers and the aim of communicating current biomedical research topics.

## Methods

**Figure 2.**
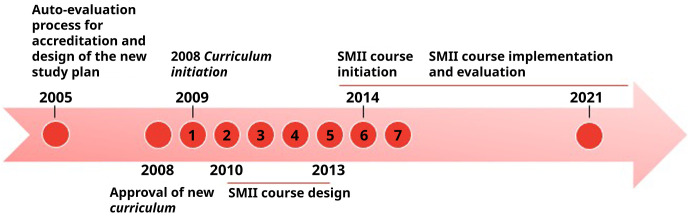
Time-flow for approval and initiation of the new *curriculum* in the School of Medicine, with incorporation of scientific methodology courses. Numbers 1-7 reflect the progression in the carreer of the 2009 generation of medical students.

The current *curriculum* for the Facultad de Medicina was designed and approved by the Council in 2008 and started to be progressively applied in March 2009 ( Figure 2).

According to the 2008 *curriculum*, the career has a duration of seven years, organized in three different modules: two initial modules of three years each, and the internship in the last year. Both modules finish with the Scientific Methodology I and II courses **,** respectively ( Figure 1).

Taking into consideration the Accreditation Report ^16^, a formal training process in scientific research throughout the career was incorporated into the new study plan; to this end, specific courses and activities for the incorporation of research skills and current medical and ICTs which are available were designed. The Scientific Methodology course I (SMI) for the first-time presents theoretical elements related to the design, methodological and ethical aspects of research, medical literature searches and biostatistics ( Table 1). The Scientific Methodology II course (SMII) in practical terms aims to complete the scientific training of future physicians at an advanced stage of their medical studies while working on specific research projects. The most significant contribution of SMII is that builds on top of the theoretical contents offered in the SMI course with the experience of an actual investigation. The research project covers all the stages from the conception of the hypothesis and design of the investigation to the final communication of the results, with the support of extensive literature searches and the power of current ICT.

**Figure 3.**
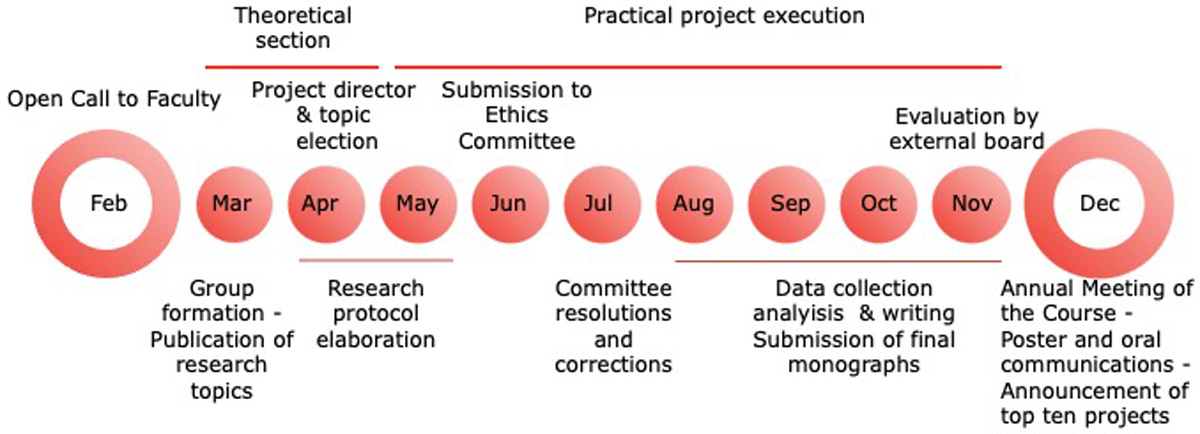
Schedule of activities in Scientific Methodology II course at Facultad de Medicina.

The SMII Course has an initial section of two months, that includes biostatistics, ICT and exploration of biomedical databases, where students learn to search in medical databases for scientific literature and are trained in the critical analysis of papers ( Table 1). This section runs in parallel with the design and execution of a research project over ten months under the supervision of Facultad de Medicina faculty ( Figure 3). 

It is noteworthy that the course is mandatory and involves the participation of 600 students and up to 300 professors *per* year. The overall course is managed and supervised by a coordinator who is a faculty member of the Facultad de Medicina and an active biomedical investigator.

The course began its activities for the first time in March 2014 ( Figure 2) and has as its main goal the design and execution of a research project in groups of six students mentored by faculty (both junior and senior). Professors are recruited each year by an open call to the faculty members to voluntarily propose a research topic in which the students will perform their investigations, depending on their own interests. The selection process is performed in the virtual campus platform where all the available topics proposed by the faculty are displayed and selected on a first-come and first-serve basis by one student delegate *per* group.

The ten-month research process starts with the project that involves setting a hypothesis and establishing the research design and corresponding protocols which must be formally presented to the corresponding ethics or animal welfare committees. Once the project is approved, the groups start to collect and analyze data followed by data interpretation, discussion and conclusions. The group and faculty members meet periodically at least twice *per* month over the year for guidance and evaluation of the process ( Figure 3).

The project finishes with the generation of: a) a monographic work by each group, with a predefined format of an original paper or review article, ^g^ and b) a two-day poster presentation in the Annual Meeting of the course, with the participation of the students and professors of Facultad de Medicina ( Figure 3). Topics proposed cover all the aspects of the medical sciences related to the different departments of the school ( *e.g.,* basic, epidemiological and clinical areas), with several projects in translational medicine.

The completed research project carried out by the students is evaluated by an external evaluating committee that selects a set of works to be published in the official journal of Facultad de Medicina, *Anales de la Facultad de Medicina*. The evaluation of all the projects is first performed using a guideline to standardize criteria, followed by a meeting of the whole committee to perform an extensive analysis of the different projects and select the best ones. The publication process allows the students to become familiar with the editorial process and represents an important aspect for the diffusion and promotion of some of the research performed in the different departments of the Facultad de Medicina.

The SMII course Annual Meeting allows the presentation of the results of all the participating groups (100 groups *per* year) and a vivid collegial exchange between students and professors.

Next, the evolution of the eight-year experience in the implementation of the SMII course will be described.

## Results

**Figure 4.**
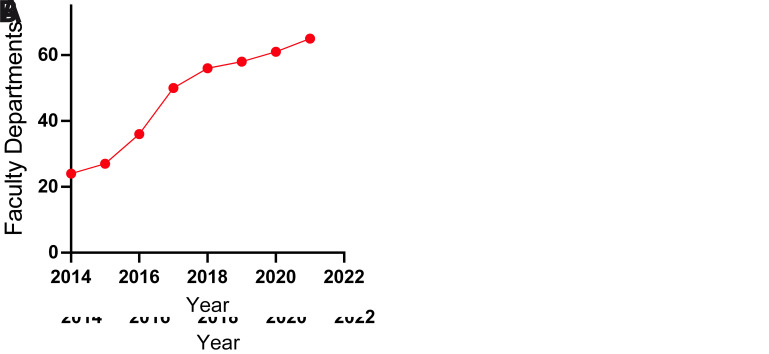
Participation of Faculty Professors as project mentors and Medical School Departments *per* year in the Scientific Methodology course (2014-2021) ^18^.

The Scientific Methodology II course implies the participation of a large number of faculty members either in their role of project directors or evaluators. Since the course was established in 2014, the number of faculty and departments involved has steadily increased, with the important participation of most of the basic, epidemiological and clinical services. In 2021, over 300 professors were involved in the direction of research projects, and around 60 departments in all basic and clinical areas, from a total of 80, participated ( Figure 4) ^18^. It is important to note that in the initial years ( *e.g.,* 2014) there was one professor allocated to each group, while now there is an average of three professors *per* group, in many cases from different departments ( *e.g.,* 100 in 2014, and 300 in 2021) which helps to foster interdisciplinarity.

**Figure 5.**
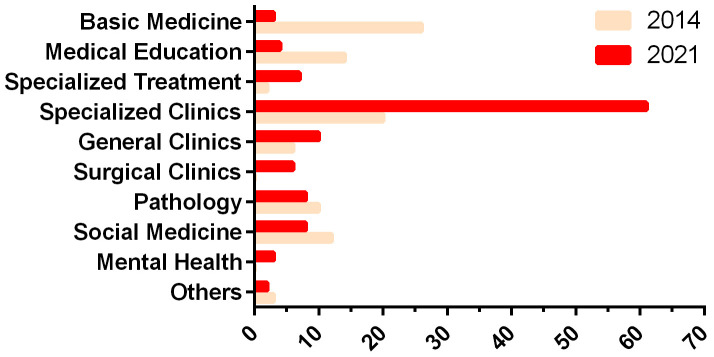
Distribution of topics by areas in SMII course (2014–2021), as classified in Facultad de Medicina, Universidad de la República ^18^.

An important issue to mention is that initially there was the significant participation of professors from the basic areas, who had considerable expertise in research activities (28% from basic medicine, 41% from all clinical areas in 2014). This changed significantly over the years and actually there is a significant involvement of the general and specialized areas which is an important goal of these courses (3% from basic medicine, 81% from all clinical areas in 2021) ( Figure 5) ^18^.

If a closer analysis of the distribution of areas of Facultad de Medicina that are participating since 2014 is done it can be observed that, since 2015, general and specialized clinical departments increased the number of mentors and projects, notably for the surgical areas, which had no participation at all in 2014, and are now participating in several groups ( Figure 5). Indeed, the SMII course nurtures novel research development in surgical areas.

Each year a diversity of topics from all departments are presented which allow students to work in different areas of their interest, since they will choose their topics at the beginning of the year, and usually are related to their future specialization.

Since the SMII course was settled, special issues of the journal *Anales de la Facultad de Medicina ^h^* were published, containing the selected publications of each year. Most of the monographs published each year are included at the *Colibrí Database* from *Universidad de la República,* a repository which contains much of the graduate and doctoral thesis and projects from Uruguay.

The list of research project topics was expanded with time, as new professors and departments became involved into the direction of projects ^18^. In some cases, professors from schools other than Medicine were involved in co-direction of the projects, further promoting an interdisciplinary approach. Many of the selected topics have been closely related each year to the social and health situation of the country, and original knowledge has been created in topics which are relevant to our society, such as the current situation and prevalence of different diseases, clinical evaluation of the use of drugs, incorporation of new diagnosis tools or novel therapies, perception of the quality of health care in public hospitals, environmental conditions, mental health and medical education learning conditions, among others. Several of these research projects are the first step for more in-depth investigations, and this is an important contribution of the SMII course to the research of Facultad de Medicina ( Table 2) ^18^. Clinical faculty are highly dedicated to assistance duties, having limited opportunities to carry on research activities, and therefore the projects performed in the context of the SMII course have had an important catalyzing role in the promotion of research activities in the different departments.

**Table 2.** Selected topics in the Scientific Methodology II course (2014–2021) and distribution among the different medical areas of Facultad de Medicina (titles translated from Spanish).

Area	Department	Research Topic	Year
**Basic Medicine**	Immunology	Cancer immunotherapy using antibodies that recognize carbohydrate antigens	2014
	Biochemistry- Neonatology	Mitochondrial Diseases: diagnostic challenge	2016
	Histology	Male infertility: diagnosis and etiopathogenesis: Role of sperm morphology	2017
**General Clinics**	Internal Medicine	Prevalence of anemia in patients assisted in the Multidisciplinary Heart Failure Unit of the Hospital de Clínicas Manuel Quintela, Universidad de la República, Uruguay: 2014–2015	2015
	Internal Medicine	Fibroscan® as a diagnosis of portal hypertension in cirrhotic patients at Hospital Pasteur, Universidad de la República in the period 2015 – 2018	2018
	Pediatrics	Treatment of Attention Deficit Hyperactivity Disorder in children and adolescents	2014
	Internal Medicine	Diabetes in heart failure: descriptive study in a Multidisciplinary Heart Failure Unit of the Hospital del Clínicas, Universidad de la República, Uruguay 2016	2016
	Pediatrics	Prevalence of gestational smoking and other addictions in infants who entered the Unexpected Infant Death program, Uruguay, 2010–2015	2016
	Internal Medicine	Characterization of patients with Systemic Lupus Erythematosus and hemolytic anemia assisted in the Systemic Autoimmune Diseases Unit of the Hospital de Clínicas, period 2016–2017	2017
	Internal Medicine	Frequency and etiology of spontaneous bacterial peritonitis in patients with liver cirrhosis: Hospital Maciel, 2016–2017, a descriptive study.	2017
	Internal Medicine	Analysis of the level of satisfaction of users of a Heart Failure Unit after an intervention, Hospital de Clínicas, Universidad de la República, Uruguay, 2015	2015
	Internal Medicine	Descriptive study of stroke patients assisted at Hospital Maciel, Universidad de la República during the period 2016–2017	2017
**Medical** **Education**	Biophysics	Evaluation of knowledge regarding the therapeutic use of cannabis in students and teachers of Facultad de Medicina, Universidad de la República, 2015.	2015
	Internal Medicine	Prevalence of Burnout Syndrome in residents of Hospital de Clínicas Dr. Manuel Quintela, Montevideo, Uruguay, 2018	2018
	Internal Medicine	Correlation between educational climate and empathy in Medicine students studying the Internship (2019–2020)	2020
**Mental Health**	Psyquiatrics	Child sexual abuse: A review of its characteristics and repercussions	2017
	Psyquiatrics	Description of suicidal behavior in users of Centro de Salud Jardines del Hipódromo, Montevideo, Uruguay, 2019	2019
**Pathology**	Basic Medicine	Flow cytometry for the monoclonal gammopathies diagnosis in Hospital de Clínicas, Universidad de la República, Uruguay, period 2014-2017	2016
	Bacteriology & Virology	Tuberculosis: epidemiology, diagnostic methods and treatment	2014
	Bacteriology & Virology	Comparative evaluation of the knowledge of medical and veterinary personnel about human leptospirosis in Uruguay, 2015	2015
	Pharmacology	Active pharmacovigilance and characterization of a population of Uruguayan users of medicinal cannabis derivatives, 2018	2018
	Bacteriology & Virology	Description of the antibiotic susceptibility profile to Escherichia *coli* isolated from urine cultures in Uruguay, 2018–2019	2019
**Social Medicine**	Social Preventive Medicine	Efficacy and safety of cannabis derivatives in the treatment of cancer-related pain: review and meta-analysis	2019
	Social Preventive Medicine	Domestic violence: approach from the established for healthcare practice in the System of Health of Uruguay	2014
	Social Preventive Medicine	Inequities in access to kidney transplantation in patients in chronic dialysis in Uruguay, period 2005–2012	2014
**Specialized** **Clinics**	Cardiology	Utility of intravascular ultrasonography (IVUS) for the evaluation of angiographically intermediate coronary lesions	2014
	Neonatology	Perinatal asphyxia: correlation between brain monitoring and mitochondrial dysfunction in a newborn pig model	2016
	Hematology	Study of Anemia in the elderly assisted in the Hospital de Clínicas, Universidad de la República, January–April 2019	2019
	Obstetrics	Prevalence of caesarean section in the maternity ward of Hospital de Clínicas, Montevideo-Uruguay, 2017	2017
	Toxicology	Association between source of exposure and severity of carbon monoxide poisoning	2018
	Intensive Medicine	Analysis of inflammatory biomarkers in burned patients with and without bacteremia	2019
**Specialized** **Treatment**	Imagenology	Evaluation of the sentinel lymph node strategy in Melanoma applied at the Nuclear Medicine Center, Hospital de Clínicas	2014
	Internal Medicine	Pulse oximetry and its utility in neonatal screening for critical congenital heart disease	2016
	Imagenology	Tomosynthesis associated with 2D digital mammography in the early detection of breast cancer	2017
	Hematology	Rational use of fresh frozen plasma and its implication in the production of blood products	2018
	Hematology	Thrombophilia study in women who had a pregnancy in the period 2011–2020 in Uruguay	2020
**Surgical Clinics**	Internal Medicine- Surgery	Thromboembolic events and thromboprophylaxis in cancer surgical patients at the Hospital de Clínicas, Universidad de la República during 2014–2016	2017
	Internal Medicine- Surgery	Descriptive study of surgical infections in emergency surgeries at Centro Hospitalario Pereira Rossell, Universidad de la República, January–July 2016	2016
	Internal Medicine- Surgery	Nutritional evaluation in patients with chest surgery at the Hospital de Clínicas during June-September 2018	2018
**Others**	Bioethics - Medical Engineering	Application of artificial intelligence in the health field: bibliographic review	2019

To exemplify, selected topics from the past eight years are shown in Table 2 and Table 3, underscoring the high diversity on research themes and areas ^18^.

**Table 3.** Coronavirus disease (COVID-19)-related topics during 2020–2021 Scientific Methodology II courses (titles translated from Spanish).

Area	Department	Research Topic	Year
**Basic Medicine**	Anatomy	International analysis of human anatomy teaching in times of COVID-19	2020
	Biophysics	Inference of epidemic models for COVID-19 in Uruguay in 2020 based on public data	2020
**General Clinics**	Internal Medicine	Immunopathogenesis of SARS-CoV-2 infection and its clinical implications	2020
	Internal Medicine	Estimation of the prevalence of SARS-CoV-2 in lupus treated with hydroxychloroquine. Hospital de Clínicas, Médica Uruguaya, 2020	2020
	Internal Medicine	Cardiovascular compromise in patients infected by SARS-CoV-2	2020
	Internal Medicine	Repercussions of the COVID-19 pandemic in a population of COPD patients at Hospital Pasteur	2021
	Pediatrics	Impact of the SARS Covid 19 pandemic on childhood sleep and early adolescence	2021
	Pediatrics	Consequences of the COVID-19 pandemic in the health care of children and adolescents in the first level of care in the public and private sector of Montevideo	2021
**Social** **Medicine**	Bioethics	Decision-making at the end of life in the context of COVID: A reflection from bioethics	2020
	Social and Preventive Medicine	Efficacy and safety of vaccines against COVID-19: bibliographic review of vaccines used in the region	2021
**Specialized ** **Clinics**	Intensive Care	Predictive factors of clinical outcomes during invasive mechanical ventilation in critically ill COVID 19 patients	2020
	Obstetrics	Perinatal outcomes of SARS-CoV-2 infected pregnant patients: a literature review	2020
	Hematology	Survey of the therapeutic approach to hemostasis alterations in patients with COVID-19	2020
	Nefrology-Infectious Diseases	Characterization of the symptoms of confirmed and suspected COVID-19 patients in a private health centre, in the period March-June 2020.	2020
	Neumology	Description of the first individuals infected by SARS-CoV-2 in Uruguay	2020
	Pediatrics	Sleep disorders in the pediatric population and its relationship with the COVID 19 pandemic, 2020	2020
	Pediatrics	Social distancing in the prevention of SARS-CoV-2: Risks and benefits of school closure	2020
	Pediatrics Emergency	Description of the effect of telephone counseling in the context of the COVID-19 pandemic at the Pediatrics Emergency, Centro Hospitalario Pereira Rossel, March-July 2020	2020
	Cardiology	Study of the incidence of out-of-hospital cardiorespiratory arrests in the period April 2020-April 2021 during the SARS-CoV-2 pandemic	2021
	Physical medicine	Rehabilitation in post-Covid 19 patients: role of telemedicine	2021
	Pediatrics- Neuropediatrics	Impact of the interruption of presence due to the SARS-CoV-2 COVID-19 pandemic on the health of children in initial education in Uruguay, 2020	2021
	Heart Surgery	Risk factors that increase the possibility of SARS-CoV-2 infection in treated patients with a history of cardiovascular disease	2021
	Emergency	Comparison of inflammatory markers in patients with pneumonia caused by SARS-CoV-2 vs patients with pneumonia caused by other pathogens	2021
	Endocrinology and Metabolism	Metabolic control, number of consultations, access to medications and exercise in patients with diabetes during the Covid-19 pandemic.	2021
	Intensive Care	Monitoring and analysis of positive COVID patients in the Intensive Care Unit of the Hospital de Clínicas Dr. Manuel Quintela	2021
	Nefrology	Anti-SARS-CoV-2 antibodies in patients with renal and renopancreas transplantation after vaccination and their persistence over time. Study of clinical and immunological factors associated with seroconversion	2021
	Obstetrics	Description of obstetric and neonatal outcomes in COVID-19 positive mothers	2021
	Neonatology	Neonatal impact of the SARS-CoV-2 pandemic on the maternity hospital at the University Hospital	2021
	Neuropediatrics	Self-perception of children in a pandemic situation due to COVID 19	2021
**Medical** **Education**	Medical Education	Study on the impact of distance learning on students of the medical clinical course of the Facultad de Medicina, Universidad de la República, in the context of the COVID-19 pandemic	2021
	Familiar Medicine	Descriptive study of the effects of the COVID-19 pandemic on tobacco and cannabis use in students, Facultad de Medicina, 2020-2021	2021
**Mental Health**	Medical Psychology- Oncology	Fatigue and compassion satisfaction in doctors and nurses in the context of the COVID-19 pandemic in Uruguay	2021
**Surgical Clinics**	Heart Surgery	Risk factors that increase the possibility of SARS-CoV-2 infection in treated patients with a history of cardiovascular disease	2021

In 2020, the coronavirus disease (COVID-19) pandemic imposed an additional challenge on all the faculty staff and on the students themselves. Despite the fact that the health situation in Uruguay was for the most part of the evolution of the pandemic significantly better than other countries of the region and the world ^19, 20^, we also had to adapt part of the course to virtual activities, and there were some periods of the year when access to the university and hospitals was restricted or even forbidden. However, this context was used by many of the groups to carry out work related to SARS-CoV-2 and COVID-19, which ended up generating a wealth of relevant local information in the 2020–2021 courses. Table 3 summarizes some of the topics related to the COVID-19 pandemic, showing the adaptation to these particular circumstances ^18^.

Last year, at the end of the 2020 course and due to the pandemic, the Annual Meeting was restructured, and the presentation of posters could not be done as usual because the university was closed at that time of the year. Instead, the 100 groups presented their work orally through a virtual platform, constituting an unprecedented and unique experience in Facultad de Medicina, which will be incorporated in future editions of the course.

In the pandemic context, it is noteworthy to note the creation of an honorary scientific advisory group ( GACH) ^i^ that worked during 2020 and until July 2021 advising the national government in making decisions based on the best available scientific evidence ^19^. This group was assembled with recognized scientists and physicians, many of whom are faculty of the School of Medicine, who at the same time worked in SMII either in the mentoring of the groups or in the evaluation of the projects. For our medical students, this was a compelling and “live” example of how appropriate scientific training can directly impact in the management and the resolution of health problems in our society.

## Conclusions

The described Scientific Methodology course (SMII) has successfully evolved after eight years of execution, resulting in one of the most important incorporations of the current 2008 *curriculum*. The students finished the course with knowledge on how to use, navigate and gather information from databases such as Pubmed, Cochrane and Lilacs, among others, understand how to formulate and progress through a small research project, consider aspects of bioethical and animal welfare and go through the approval process, get trained in scientific reading and English language, collect and interpret data, generate a complete written document, and communicate in public their work in poster format. All of the participating parts and international faculty have qualified this activity as an educational breakthrough in our institution. Students and professors consider this course as a highly gratifying and productive experience. An additional positive outcome was the “reactivation” of some research topics in the departments, in a way that largely exceeded the boundaries of the course. Also, some departments which were not significantly involved in research activities started to participate actively in the course, and generate new research lines in their areas. In addition, some of the students were integrated in research groups and communicated their work at local and international meetings, publishing their investigations in national and international journals in an early stage of their career. This action is now synergizing with other courses of the *curriculum* with the final aim to incorporate the scientific methodology approach as a continuous process through the medical career. Another outcome of the course is the participation of professors as co-directors from other faculties ( *e.g.,* Sciences, Psychology and Engineering), generating interdisciplinary projects around the university.

In addition, Uruguayan scientific and medical societies have included presentations from the SMII course students in their meetings, and many research projects have resulted in original publications both in national and international journals.

In spite of the great number of students of each generation, an innovative and successful program was instated in our institution. The course impacted positively on the scientific background of advanced medical students, and renovated the research activities in many clinical Departments.

Recently, a similar experience was published by Uebel *et al.* at New South Wales University, Australia, however, the number of students involved is much lower than the one of Facultad de Medicina, Universidad de la República. They reported the implementation of an independent learning project (ILP), to promote research skills within the medical students in the whole cohort of the last year of the career where students performed a 34-week research project. Similar to what is reported in this paper, they conducted a long evaluation process (14 years), concluding that students gained valuable experience in research methodology ^21^.

Though similar experiences are incorporated in different medical schools around the world, it is remarkable that Facultad de Medicina has a quite large number of students at this stage of their medical studies ( *i.e.*, 600 students). This condition represents an additional challenge since the proposal intends to allow the students to undergo all the stages of a research project in a short period of time (10 months).

While this manuscript describes the background, design, execution and direct outcomes of the course, a quantitative study of its impact in the incorporation of research skills and scientific performance of graduate medical students in different cohorts of Facultad de Medicina (2010–2021) is now being conducted. These data will allow to provide further objective elements on the influence that incorporation of formal scientific training in the *curriculum* of medical students has on continuous education and professional performance. In line with Flexner’s original views on the role of research training in medical education, the SMII course experience is providing us with positivity and hope in the context of the future medical practice and also on how medical research is perceived by physicians as an integral part of the health system.

## Data availability

Zenodo: Scientific Methodology Course II-Facultad de Medicina, Universidad de la República, Uruguay (2014–2022). https://doi.org/10.5281/zenodo.6625343 ^18^

This project contains the following underlying data:

- Scientific Methodology Course-Facultad de Medicina-Universidad de la República-2014–2022.csv (General data from the course, period 2014–2021)
- SMC-Research Topics-2014.csv (Research projects year 2014)
- SMC-Research Topics-2015.csv (Research projects year 2015)
- SMC-Research Topics-2016.csv (Research projects year 2016)
- SMC-Research Topics-2017.csv (Research projects year 2017)
- SMC-Research Topics-2018.csv (Research projects year 2018)
- SMC-Research Topics-2019.csv (Research projects year 2019)
- SMC-Research Topics-2020.csv (Research projects year 2020)
- SMC-Research Topics-2021.csv (Research projects year 2021)

Data are available under the terms of the Creative Commons Attribution 4.0 International license (CC-BY 4.0).

## Footnotes

^a^ “Facultad de Medicina” will be the term used throughout the manuscript to indicate the School of Medicine of Universidad de la República, Uruguay

^b^ The first three years of medical school at Universidad de la República are equivalent to that of undergraduate training in the USA, as Uruguayan students start at the Facultad de Medicina right after high school. The clinical work at the hospitals starts in the 4 ^th^ year of training.

^c^ Asamblea del Claustro, translated from Spanish as *Cloister Assembly*, is part of the government structure of each School at Universidad de la República, which is constituted by elected members, representing faculty, students and professionals. Universidad de la República is an autonomous and co-governed entity as defined in the National Constitution; ruled by the “Ley Orgánica 12,549” from 1968.

^d^ The “MERCOSUR region encompasses the “southern cone” countries of South America, Argentina, Brazil, Paraguay and Uruguay.

^e^ By its initials in Spanish: *Mecanismo Experimental de Acreditación de Carreras;* translated as *Experimental Mechanism for Accreditation of Careers.*

^f^ By its initials in Spanish: *Sistema de Acreditación Regional de Carreras Universitarias del MERCOSUR;* translated as *Regional Accreditation System of University Careers of MERCOSUR*. ARCU-SUR includes educational agreements between MERCOSUR member countries (Uruguay, Brazil, Argentina and Paraguay) and associated member countries (Chile and Bolivia).

^g^ A guideline is provided so that all monographs have preestablished sections, format and length and must include a given number of tables, figures and references.

^h^ Official journal of Facultad de Medicina, AnFaMed - ISSN 2301-1254, indexed in SciELO

^i^ GACH from its initials in Spanish, Grupo Asesor Científico Honorario; the General Coordinator of the group was one of the coauthors, Rafael Radi, MD, PhD.

## References

[1] Spencer AL Brosenitsch T Levine AS : Back to the basic sciences: an innovative approach to teaching senior medical students how best to integrate basic science and clinical medicine. *Acad Med.* 2008;83(7):662–9. 10.1097/ACM.0b013e318178356b 18580085

[2] Universidad de la República: Plan estratégico de desarrollo de la Universidad de la República.2020;376. Reference Source

[3] Flexner A : Medical Education in the United States and Canada.The Carnegie Foundation for the Advancement of Teaching; Bulletin Number Four.1910. Reference Source PMC2567554 12163926

[4] Medawar SP : Scientific method in science and medicine. *Perspect Biol Med.* 1975;18(3):345–52. 10.1353/pbm.1975.0030 1196837

[5] Norman G : The essential role of basic science in medical education: the perspective from psychology. *Clin Invest Med.* 2000;23(1):47–51; discussion 52–4. 10782317

[6] Cooke M Irby DM Sullivan W : American medical education 100 years after the Flexner report. *N Engl J Med.* 2006;355(13):1339–44. 10.1056/NEJMra055445 17005951

[7] Fleming KA : Flexner at 100: a brief view from Oxford. *Perspect Biol Med.* 2011;54(1):24–9. 10.1353/pbm.2011.0013 21399380

[8] Ludmerer KM : Commentary: Understanding the Flexner report. *Acad Med.* 2010;85(2):193–6. 10.1097/ACM.0b013e3181c8f1e7 20107341

[9] Ludmerer KM : Abraham Flexner and medical education. *Perspect Biol Med.* 2011;54(1):8–16. 10.1353/pbm.2011.0009 21399378

[10] Services CoGME-DoHaH: Preparing learners for practice in a managed care environment.1997;1–50.

[11] Grande JP : Training of physicians for the twenty-first century: role of the basic sciences. *Med Teach.* 2009;31(9):802–6. 10.1080/01421590903137049 19811184

[12] Huynh V Christian N Tuthill K : Development of a Surgical Research Program for Medical Students and its Short-Term Impact on Academic Productivity. *J Surg Educ.* 2021;78(6):e68–e71. 10.1016/j.jsurg.2021.06.014 34266791

[13] Waaijer CJF Ommering BWC van der Wurff LJ : Scientific activity by medical students: the relationship between academic publishing during medical school and publication careers after graduation. *Perspect Med Educ.* 2019;8(4):223–9. 10.1007/s40037-019-0524-3 31290118 PMC6684557

[14] Laskowitz DT Drucker RP Parsonnet J : Engaging students in dedicated research and scholarship during medical school: the long-term experiences at Duke and Stanford. *Acad Med.* 2010;85(3):419–28. 10.1097/ACM.0b013e3181ccc77a 20182114

[15] Stead WW Searle JR Fessler HE : Biomedical informatics: changing what physicians need to know and how they learn. *Acad Med.* 2011;86(4):429–34. 10.1097/ACM.0b013e3181f41e8c 20711055

[16] Universidad de la República: Informe de Autoevaluación Institucional Facultad de Medicina.Universidad de la República, Uruguay.2012. Reference Source

[17] Facultad de Medicina: Mercosur Educativo: Dimensiones, Componentes, Criterios e Indicadores para la Acreditación del MERCOSUR.Universidad de la República, Uruguay;2010. Reference Source

[18] Bartesaghi S Radi R : Scientific Methodology Course II-Facultad de Medicina, Universidad de la República, Uruguay (2014-2022).(1.0) [Data set]. Zenodo.2022. 10.5281/zenodo.6625343

[19] Haldane V De Foo C Abdalla SM : Health systems resilience in managing the COVID-19 pandemic: lessons from 28 countries. *Nat Med.* 2021;27(6):964–80. 10.1038/s41591-021-01381-y 34002090

[20] Taylor L : Uruguay is winning against covid-19. This is how. *BMJ.* 2020;370:m3575. 10.1136/bmj.m3575 32948599

[21] Uebel K Pervaz Iqbal M Adelstein BA : A pragmatic approach to promoting research skills in all medical students. *Med Educ.* 2020;54(5):445–46. 10.1111/medu.14097 32173895

