# Supplementary material for: A scientific methodology course for advanced medical students: an eight-year perspective
Source: MedEdPublish (2016). 2022 Jul 6;12:50. [Version 1] doi: 10.12688/mep.19171.1 (PMC9427080; doi:10.12688/mep.19171.1)
Supplement: Peer review history

# Peer review: Reviewer response for version 1

**DOI:** 10.21956/mep.20536.r32420

**Published:** 2022-08-18

**Authors:** Erik Cobo

**Affiliations:** 1 Department of Statistics and Operations Research, Universitat Politècnica de Catalunya, Barcelona, Spain

I have read, and enjoyed, the paper “A scientific methodology course for advanced medical students: an eight-year perspective”.

 First at all, let me apologize for my poor English. Anyway, the paper is easy to read. At least, to me.

 This paper describes nicely an interesting learning experience, without major statements that would require formal statistical inference. I do not have major concerns. Only minors, that the authors might address or not. Please note I call those minors, as ‘comments’, not suggestions.

 So, I think it deserves indexing.

 Minor comments.

 In the introduction you stated “… fosters a focused training of medical students in scientific aspects of the medical sciences…”. I’m not sure I agree with this “general” statement. Please, clarify what are you proposing. [For instance, I think, general medical students, who will get a “medical practice” degree, should (1) recognize the major hierarchy of reproducible science over personal believes and (2) be trained to incorporate unbiased research results into their practical clinical guides. I’m not sure if this requires a full understanding of sophisticated statistical methodologies, such us the “out of date” P-value --see the ASA statement].

 In the next paragraph you refer to “advanced” students. Please clarify: how do you define ‘advanced’ in operational terms? [In my first reading, I erroneously thought this subject was optional!]

 Lastly, you stated “early participation in research can influence the long-term scientific activities of clinicians”. It seems to me that your objective is to train practice medicine physicians in order to select the best ones to do research. In my opinion this is a spurious objective. [As we do not have an easy way to compute a measurement of the physician performance, we promote them according their impact factor, assuming a strong correlation between IF and clinical performance. Unproven and dangerous assumption - I think! I also wonder if requiring research for clinical promotion have contributed to the 85% waste of research investment. ^1^ Furthermore, what proportion of publications have any value to improve clinical practice?]

 Please consider diminishing overlapping of the second paragraph methods with previous wording.

 Page 6, first paragraph. You require students to start stating their “hypothesis”. I’m overwhelmed by the extensive use of the word “hypothesis” and I wonder if this will remain in the future, after the criticisms to the P-value and the abuse of “significant” ^2^. As an example, your own paper, had any concrete hypothesis? Might you describe your aim without using the term ‘hypothesis’? In addition, please consider deleting the term ‘significant’.

 You use “fashion” terms such as personalized and translational Medicine. I wonder if they are needed to your report. [Personally, I think each intervention is already “personalized” with the definition of the eligibility criteria. ^3^]

 Please, consider starting your results with a demographical description of your subjects, both students and trainers.

 Please, consider reporting frequencies including their numerator and denominator. For example, “X out of n (28%) departments from basic medicine” instead of “28% from basic medicine”.

 I also wonder if the statistical principles used in pharmacology (such as a constant effect) apply to surgery, because a different effect can be assumed to each interventionist - see the consort extension to non-pharmacological interventions. So, although I like the implication of surgeons and other non-pharmacological experts in this project, I wonder if they have to learn the same statistical methods.

 You start the conclusions section stating the “success” of your training. I think you can be proud, but I have been trained to strictly define the rules of a “success” before starting. Please, consider describing in the previous results section the indicators that would allow you this statement - specifying you have done this once you have viewed the results and your paper is not free of the risk of bias for selective reporting.

 Other comments

 I liked your proposal in footnote ‘a’ to call “Facultad de Medicina” your school. Please review you use this term throughout the text, figures and footnotes.

Is the rationale for developing the new method (or application) clearly explained?

Yes

Is the description of the method technically sound?

Yes

Are the conclusions about the method and its performance adequately supported by the findings presented in the article?

Yes

If any results are presented, are all the source data underlying the results available to ensure full reproducibility?

Yes

Are sufficient details provided to allow replication of the method development and its use by others?

Yes

Reviewer Expertise:

Statistical methods, clinical trials, reporting guidelines

I confirm that I have read this submission and believe that I have an appropriate level of expertise to confirm that it is of an acceptable scientific standard.

## References

[1] : Avoidable waste in the production and reporting of research evidence. *The Lancet* .2009;374(9683) : 10.1016/S0140-6736(09)60329-9 86-89 10.1016/S0140-6736(09)60329-9 19525005

[2] : Scientists rise up against statistical significance. *Nature* .567(7748) : 10.1038/d41586-019-00857-9 305-307 10.1038/d41586-019-00857-9 30894741

[3] : Does evidence support the high expectations placed in precision medicine? A bibliographic review. *F1000Res* .2018;7: 10.12688/f1000research.13490.5 30 10.12688/f1000research.13490.5 31143439PMC6524747

# Peer review: Reviewer response for version 1

**DOI:** 10.21956/mep.20536.r32371

**Published:** 2022-08-16

**Authors:** Jason Booza

**Affiliations:** 1 Department of Family Medicine and Public Health Sciences, Wayne State University, Detroit, MI, USA

This manuscript provides a detailed case study of how an a large, well-established medical school can design, implement and evaluate a scientific methodology course. Overall, the authors were able to demonstrate the success of the program through a eight year retrospective analysis. The authors provide an extensive background that not only includes of narrative of their local progression towards the development of the course, but also an international perspective as well. The authors also provide sufficient detail on the development of the course and its initial implementation. Through the data analysis, they are able to demonstrate the growth of the program and the success through successful dissemination, including publication, of student projects. Overall, the manuscript is well written and addresses an important topic in medical education.

Is the rationale for developing the new method (or application) clearly explained?

Yes

Is the description of the method technically sound?

Yes

Are the conclusions about the method and its performance adequately supported by the findings presented in the article?

Yes

If any results are presented, are all the source data underlying the results available to ensure full reproducibility?

Yes

Are sufficient details provided to allow replication of the method development and its use by others?

Yes

Reviewer Expertise:

Medical Education Research, Program Evaluation, Accreditation

I confirm that I have read this submission and believe that I have an appropriate level of expertise to confirm that it is of an acceptable scientific standard.

# Response

**Published:** 2022-08-16

**Authors:** Rafael Radi

**Affiliations:** Universidad de la República, Uruguay

I thank the insightful and detailed comments of Dr. Jason Booza and I am glad for the positive appreciation and approval of our manuscript. On behalf of my coauthors, Prof. Dr. Rafael Radi
